# Melatonin attenuates fluoride-induced neurotoxicity and cognitive dysfunction through modulation of oxidative stress, neuroinflammation, and SIRT1 signaling in Wistar rats

**DOI:** 10.14202/vetworld.2026.2650-2666

**Published:** 2026-06-28

**Authors:** Newly Bagang, Nitesh Kumar, Somasish Ghosh Dastidar, Anoop Kishore, K. G. Mohandas Rao, G. Sivakumar, Smita Shenoy

**Affiliations:** 1 Department of Pharmacology, Kasturba Medical College, Manipal Academy of Higher Education, Manipal 576104, India; 2 Department of Pharmacology and Toxicology, National Institute of Pharmaceutical Education and Research (NIPER), Hajipur, Vaishali 844102, Bihar, India; 3 Centre for Molecular Neurosciences, Department of Anatomy, Kasturba Medical College, Manipal Academy of Higher Education, Manipal 576104, India; 4 Department of Pharmacology, Manipal College of Pharmaceutical Sciences, Manipal Academy of Higher Education, Manipal 576104, India; 5 Division of Anatomy, Department of Basic Medical Sciences, Manipal Academy of Higher Education, Manipal 576104, India; 6 Department of Physiology, Kasturba Medical College, Manipal Academy of Higher Education, Manipal 576104, India

**Keywords:** cognitive dysfunction, fluoride neurotoxicity, melatonin, neuroinflammation, neuroprotection, oxidative stress, SIRT1, Wistar rats

## Abstract

**Background and Aim:**

Chronic fluoride exposure causes neurotoxicity, oxidative stress, neuroinflammation, and cognitive impairment, posing a significant public health concern. This study investigated the neuroprotective potential of melatonin against fluoride-induced neurotoxicity and cognitive dysfunction in male and female Wistar rats, with emphasis on oxidative stress, neuroinflammation, apoptosis, and sirtuin 1 (SIRT1) signaling.

**Materials and Methods:**

Four-week-old Wistar albino rats (n=8 per group per sex) were exposed to sodium fluoride (NaF, 50 ppm in drinking water) alone or co-treated with melatonin (10 or 20 mg/kg, oral) for 8 weeks. Cognitive function was assessed using the Morris Water Maze (MWM) and Novel Object Recognition Test (NORT). Serum fluoride, brain SIRT1, oxidative stress markers (malondialdehyde [MDA], superoxide dismutase [SOD]), apoptosis markers (caspase-3, B-cell lymphoma 2 [Bcl-2]), inflammatory cytokines (tumor necrosis factor-α [TNF-α], interleukin-6 [IL-6]), acetylcholinesterase (AChE) levels, and hippocampal histopathology were evaluated.

**Results:**

NaF exposure significantly elevated serum fluoride, reduced brain SIRT1, increased oxidative stress, apoptosis, neuroinflammation, AChE activity, and caused hippocampal neuronal damage, leading to impaired learning and memory in both sexes (p < 0.05). Melatonin co-treatment (both doses) significantly attenuated these changes by lowering serum fluoride, restoring SIRT1 levels, reducing MDA, caspase-3, TNF-α, IL-6, and AChE, while increasing SOD and Bcl-2. It also improved behavioral performance in the MWM and NORT and preserved hippocampal neuronal morphology. Effects were comparable between sexes and between the two melatonin doses.

**Conclusion:**

Melatonin effectively mitigates fluoride-induced neurotoxicity and cognitive dysfunction in Wistar rats by modulating oxidative stress, neuroinflammation, apoptosis, and SIRT1 signaling. These findings highlight melatonin as a promising neuroprotective agent against environmental fluoride toxicity, with potential translational relevance for fluorosis-endemic areas.

## INTRODUCTION

Fluoride is a highly reactive element that occurs naturally in the environment, including air, soil, and drinking water [1, 2]. According to World Health Organization guidelines, the fluoride concentration in safe drinking water should not exceed 1.5 mg/L [3]. At optimal concentrations, fluoride has been widely used for decades to prevent and control dental caries [4]. However, excessive and prolonged fluoride exposure is associated not only with well-documented dental and skeletal fluorosis but also with adverse effects on multiple vital organs, including the brain, kidney, and liver [5]. In recent years, increasing attention has been paid to the neurotoxic potential of fluoride and its effects on the central nervous system (CNS) [6, 7].

Epidemiological studies suggest that fluoride exposure is associated with lower intelligence quotient (IQ) scores in children and an increased risk of dementia in adults [8, 9]. Some evidence also indicates possible sex-specific differences in fluoride-induced neurotoxicity, as demonstrated by prospective birth cohort studies from Canada that reported associations between higher maternal urinary fluoride concentrations and lower IQ scores in boys but not in girls, with similar observations reported in several studies from China [10, 11]. In contrast, findings from a Mexican cohort did not demonstrate this sex specificity [12].

Fluoride-induced neurotoxicity is increasingly recognized as a multifactorial process, with oxidative stress and mitochondrial dysfunction consistently identified as central pathogenic mechanisms [13, 14]. SIRT1, a nicotinamide adenine dinucleotide (NAD^+^)-dependent deacetylase highly expressed in neurons, has emerged as a key regulator of neuronal survival and brain resilience [15]. By modulating mitochondrial function, redox balance, autophagy, and neuroinflammatory signaling, SIRT1 exerts broad neuroprotective effects through several downstream molecular pathways [16, 17]. Studies suggest that excessive fluoride exposure downregulates SIRT1, leading to mitochondrial impairment, increased oxidative stress, neuronal apoptosis, and subsequent deficits in learning and memory [18, 19]. Converging evidence from both *in vivo* rat models and *in vitro* neuronal cell studies strengthens the association between SIRT1 downregulation and fluoride-induced neurotoxicity [20, 21]. These findings highlight SIRT1 dysregulation as a potential mechanistic driver of fluoride-induced neurotoxicity and support further investigation of SIRT1-targeted therapeutic interventions.

Melatonin is a neurohormone primarily produced by the pineal gland during the dark phase of the circadian rhythm [22]. Beyond its role in regulating body temperature, metabolism, sleep–wake cycles, and seasonal reproduction, melatonin has been recognized as a pleiotropic molecule with broad biological activities, including antioxidant, anti-apoptotic, anti-inflammatory, and immunomodulatory effects [23–26]. Exogenous melatonin supplementation has been widely reported to exert neuroprotective effects in various neurodegenerative conditions [27–29]. In fluoride-induced neurotoxicity, previous studies have demonstrated that melatonin confers protection mainly through its antioxidant and anti-inflammatory properties in rat models [30, 31]. Recent reports further suggest that these protective effects may involve mitochondrial regulatory mechanisms and other sirtuin-associated pathways in prenatal exposure models [32, 33]. SIRT1-mediated neuroprotection has also been documented in other experimental conditions, including lipopolysaccharide-induced oxidative stress, amyloid β toxicity, and amyotrophic lateral sclerosis [28, 29, 34].

Despite these advances, important knowledge gaps remain. Although fluoride-induced neurotoxicity has been linked to oxidative stress, mitochondrial dysfunction, and neuronal apoptosis, the contribution of SIRT1 to the protective effects of melatonin in postnatal fluoride exposure remains poorly characterized. Most previous studies have focused primarily on oxidative and inflammatory pathways, without comprehensively evaluating the relationships among melatonin treatment, SIRT1 signaling, neuroinflammation, apoptosis, and cognitive outcomes within a single experimental framework. Furthermore, available evidence regarding sex-dependent susceptibility to fluoride-induced neurotoxicity remains inconsistent. While some epidemiological investigations suggest greater vulnerability in males, others report no significant sex-related differences [10–12]. Experimental studies directly comparing male and female responses to fluoride exposure and melatonin intervention remain limited. Consequently, the extent to which biological sex influences fluoride-induced neurotoxicity and treatment responsiveness remains unclear.

Given that chronic fluoride exposure continues to pose a major public health concern in fluorosis-endemic regions, there is a pressing need to identify interventions that are effective, affordable, and readily accessible [35, 36]. Melatonin possesses several characteristics that support its translational potential, including a favorable safety profile, widespread availability, and multiple neuroprotective mechanisms. However, further experimental evidence is required to clarify its therapeutic value against fluoride-induced neurotoxicity and to better understand the molecular pathways underlying its protective effects.

Therefore, the present study was designed to investigate the neuroprotective efficacy of melatonin against fluoride-induced neurotoxicity and cognitive impairment following postnatal fluoride exposure in Wistar rats. Particular emphasis was placed on evaluating the potential involvement of SIRT1 signaling together with oxidative stress, neuroinflammation, apoptosis, and hippocampal histopathological alterations. In addition, both male and female animals were included to assess possible sex-related differences in susceptibility to fluoride-induced neurotoxicity and responsiveness to melatonin treatment. By addressing these gaps, this study aims to provide mechanistic insights into the neuroprotective actions of melatonin and to contribute to the development of potential strategies to reduce the neurological consequences of environmental fluoride exposure. The present study also aligns with United Nations Sustainable Development Goal 3 (Good Health and Well-Being) by addressing neurotoxic risks associated with environmental fluoride exposure and exploring a potential neuroprotective intervention.

## MATERIALS AND METHODS

### Ethical approval

The experimental protocol was reviewed and approved by the Institutional Animal Ethics Committee, Kasturba Medical College, Manipal Academy of Higher Education, Manipal, Karnataka, India (Approval No. IAEC/KMC/16/2020). All animal experiments were conducted in accordance with the guidelines of the Committee for Control and Supervision of Experiments on Animals, Government of India. The study adhered to accepted principles for the ethical use of laboratory animals and the 3Rs (Replacement, Reduction, and Refinement) framework. Animals were monitored daily for general health status, body weight changes, and signs of pain or distress throughout the experimental period. Humane endpoints were predefined to minimize animal suffering; however, none of the animals met the criteria for early euthanasia during the study. No protocol was preregistered before study initiation.

### Study period and location

The study was conducted from September 2022 to September 2025 at Kasturba Medical College, Manipal Academy of Higher Education, Manipal, Karnataka, India. Animal experimentation was performed at the Central Animal Research Facility, where all animals were housed and maintained under controlled environmental conditions. Behavioral assessments, biochemical analyses, and histopathological evaluations were conducted in the respective institutional laboratories following completion of the treatment period.

### Materials

Sodium fluoride (NaF; Sisco Research Laboratories Private Limited, Mumbai, India; Cat. No. 29821) and melatonin (Tokyo Chemical Industry (India) Private Limited, Chennai, India; Cat. No. M1105) were used in this study. Enzyme-linked immunosorbent assay (ELISA) kits were procured from Krishgen Biosystems Private Limited, Mumbai, India, including Rat Sirtuin 1 (Cat. No. KLR1214), Rat malondialdehyde (MDA; Cat. No. KLR4879), Rat superoxide dismutase (SOD; Cat. No. KLR0168), Rat B-cell lymphoma 2 (Bcl-2; Cat. No. KLR0037), Rat Caspase-3 (Cat. No. KLR1648), Rat tumor necrosis factor-alpha (TNF-α; Cat. No. KLR5781), Rat interleukin-6 (IL-6; Cat. No. KLR4747), and Rat acetylcholinesterase (AChE; Cat. No. KLR0724). All kits were validated for use with rat samples and their corresponding analytes.

### Animals

Four-week-old male and female Wistar albino rats weighing 65–100 g were obtained from the in-house breeding colony of the Central Animal Research Facility, Manipal, India. Both sexes were included to assess sex-related differences in fluoride-induced neurotoxicity and treatment responses. Animals were housed under controlled environmental conditions (20–25°C, relative humidity 40%–70%, and a 12 h light/dark cycle) with ad libitum access to standard pellet diet and drinking water. Animals were maintained under standardized pathogen-controlled conditions with routine veterinary monitoring and health surveillance. No experimental procedures had been performed on the animals prior to study initiation.

### Study design

Following a 1-week acclimatization period, rats were stratified according to sex and randomly allocated to six treatment groups within each sex based on body weight. Exclusion criteria included signs of illness, distress, or significant weight loss. The individual animal was considered the experimental unit.

The treatment groups were as follows:

1. Control group: Drinking water containing ≤0.5 ppm fluoride.
2. NaF group: NaF 50 ppm in drinking water.
3. Melatonin (MLT) 10 group: Melatonin 10 mg/kg by oral gavage.
4. MLT 20 group: Melatonin 20 mg/kg by oral gavage.
5. NaF + MLT 10 group: NaF 50 ppm plus melatonin 10 mg/kg.
6. NaF + MLT 20 group: NaF 50 ppm plus melatonin 20 mg/kg.

The study used the minimum feasible group size (n = 8 per group) to comply with ethical principles while maintaining adequate statistical power. All animals (n = 8 per group) underwent behavioral assessments. Brain tissues from a subset of animals were used for biochemical analyses (n = 5 per group) and histopathological evaluation (n = 3 per group). Serum samples (n = 6 per group) were collected for fluoride estimation.

Melatonin solutions were freshly prepared daily and protected from light. Melatonin was dissolved in a minimal volume of absolute ethanol and subsequently diluted in drinking water. The final ethanol concentration in the administered solution was 0.006%, which was maintained in all experimental groups, including the vehicle control group [37]. Treatments were administered for 8 consecutive weeks, with melatonin administered once daily between 3:00 PM and 4:00 PM.

The NaF dose (50 ppm; approximately 3.78 mg/kg) was selected based on previous studies demonstrating reproducible neurotoxic effects, including oxidative stress, apoptosis, neuroinflammation, and cognitive impairment [21, 38–40]. Rodents have been reported to require approximately four- to five-fold higher fluoride exposure than humans to achieve comparable plasma concentrations and biological responses [41–43]. The environmental relevance of the selected exposure level is supported by reported fluoride concentrations in non-thermal environmental waters (0.04–8.3 mg/L) and in groundwater sources from fluorosis-endemic regions (3.3–11.3 mg/L) [44, 45]. The melatonin doses (10 and 20 mg/kg) were selected based on previous studies demonstrating significant neuroprotective effects in experimental models of neurotoxicity [46–48].

### Sample collection and post-treatment procedures

At the end of the treatment period, blood samples were collected from the retro-orbital plexus. Animals subsequently underwent behavioral assessments. Following completion of behavioral testing, animals were euthanized by carbon dioxide exposure in accordance with institutional animal care guidelines. Brains were immediately dissected and preserved at −80°C for biochemical analyses or fixed in 10% formalin for histopathological examination. Investigators responsible for behavioral assessments, biochemical analyses, and histopathological evaluations were blinded to treatment allocation.

**Behavioral testing:** Behavioral assessments were conducted sequentially, with the Morris Water Maze (MWM) performed before the Novel Object Recognition Test (NORT). A 48 h interval was maintained between tests to minimize potential carryover effects. Behavioral scoring was performed by investigators blinded to group allocation. Animals were acclimatized to the testing environment for at least 30 min before testing.

**MWM:** Spatial learning and memory were assessed using the MWM with minor modifications to the original method described by Morris [18, 49]. The apparatus consisted of a circular pool tank (Ugo Basile Drum, Ugo Basile, Gemonio, Italy), an escape platform, and recording equipment. The pool was divided into four equal quadrants, with the hidden platform positioned in quadrant 4. Water temperature was maintained at 26 ± 1°C and water depth at 30 cm. Milk powder was added to render the platform invisible. The platform was submerged 2 cm below the water surface.

The testing protocol consisted of 4 days, including acquisition training on days 1–3 and a probe trial on day 4. During acquisition training, each animal completed four trials/day with 15–20 min intervals between trials. Escape latency was recorded during each trial. Animals unable to locate the platform within 90 s were guided to the platform and allowed to remain there for 15 s. During the probe trial, the platform was removed and animals were allowed to explore the pool for 60 s. Escape latency, time spent in the target quadrant, platform-crossing number, and swimming speed were recorded using ANY-maze software version 7.46 (Stoelting Co., Wood Dale, IL, USA).

**NORT:** Recognition memory was assessed using the NORT in a field box measuring 100 × 100 × 60 cm over 3 consecutive days. Animals were habituated to the arena on day 1, exposed to two identical familiar objects on day 2, and presented with one familiar and one novel object on day 3. Object placement was counterbalanced among animals.

Exploration behavior was recorded for 5 min using ANY-maze software version 7.45 (Stoelting Co.). Animals were required to achieve a minimum cumulative exploration time of 20 s across both testing sessions to be included in the analysis. Exploration was defined as sniffing, touching, or rearing toward an object within a distance of 2 cm.

The discrimination index (DI) and recognition index (RI) were calculated as follows [1, 50, 51]:

DI = (Time spent exploring novel object − Time spent exploring familiar object)/(Time spent exploring novel object + Time spent exploring familiar object)

RI = Time spent exploring novel object/(Time spent exploring novel object + Time spent exploring familiar object)

**Brain tissue processing for ELISA:** Brain tissue samples stored at −80°C were thawed on ice, weighed, and homogenized using a tissue homogenizer (Telematic and Biomedical Services Pvt. Ltd., Chennai, India) in cold phosphate-buffered saline (PBS; pH 7.4) at a 1:10 (w/v) ratio to obtain a 10% tissue homogenate. The homogenates were centrifuged at 845 × *g* for 20 min at 4°C, and the resulting supernatants were aliquoted and stored at −80°C until analysis. Sample processing was performed according to the manufacturer’s instructions for each ELISA kit. Analyte concentrations were calculated from standard curves and expressed as ng/mL or pg/mL in wet tissue homogenates.

**Measurement of serum fluoride:** Blood samples were collected from the retro-orbital plexus using capillary tubes [52, 53]. Following centrifugation at 845 × *g* for 10 min at 4°C using a refrigerated centrifuge (REMI C-24 Plus, REMI Elektrotechnik Ltd., Mumbai, India), serum was separated and collected for analysis. Serum fluoride concentrations were determined using the ion-selective electrode method with an Orion Star A214 Fluoride Meter (Thermo Fisher Scientific, Waltham, MA, USA). The electrode was calibrated before use and recalibrated at regular intervals (every 3–4 days) using fluoride standards. The measurable detection range of the instrument was 0–999 ppm. Serum fluoride concentrations were measured to confirm fluoride exposure and evaluate systemic fluoride status among experimental groups.

**Measurement of brain SIRT1:** Brain SIRT1 concentrations were quantified using the Rat SIRT1 GENLISA™ ELISA kit (Krishgen Biosystems) according to the manufacturer’s instructions. Briefly, 100 μL of standards, tissue homogenates, and assay reagents were added to each well as specified in the protocol. Absorbance was measured using a Multiskan SkyHigh microplate spectrophotometer (Thermo Fisher Scientific). All samples were analyzed in triplicate. The assay precision reported by the manufacturer indicated an intra-assay coefficient of variation (CV) of <8% and an inter-assay CV of <10%.

**Assessment of oxidative stress and apoptosis markers:** Brain concentrations of MDA, SOD, caspase-3, and Bcl-2 were determined using commercially available ELISA kits (Krishgen Biosystems). All assays were performed according to the manufacturer’s instructions without modification. Samples were analyzed in triplicate. The reported assay precision was an intra-assay CV of <8% and an inter-assay CV of <10%.

**Assessment of inflammatory cytokines and AChE:** Brain concentrations of TNF-α, IL-6, and AChE were quantified using commercially available ELISA kits (Krishgen Biosystems). All assays were performed strictly according to the manufacturer’s instructions without procedural modifications. Samples were analyzed in triplicate. The reported assay precision was an intra-assay CV of <10% and an inter-assay CV of <12% for TNF-α and IL-6, whereas AChE assays demonstrated an intra-assay CV of <8% and an inter-assay CV of <10%.

### Histopathology of the hippocampus

Following euthanasia, brains were collected after cardiac perfusion and fixed in 10% neutral-buffered formalin. After fixation for 24–48 h at 4°C, tissues were dehydrated, embedded in paraffin, and sectioned at 8 μm thickness according to standard histological procedures [54, 55]. Following deparaffinization and rehydration through graded alcohols, sections were stained with 0.1% cresyl violet solution (LOBA Chemie Pvt. Ltd., Mumbai, India) for Nissl staining [54].

Morphological alterations in the hippocampal regions, including Cornu Ammonis 1 (CA1), Cornu Ammonis 3 (CA3), and dentate gyrus (DG), were examined using a Nikon Eclipse E200 light microscope (Nikon Corporation, Tokyo, Japan). Images were captured at 10× and 40× objective magnifications.

Quantitative neuronal assessment was performed in the CA1, CA3, and DG regions with minor modifications to the method described by Farhat *et al*. [54]. Images containing a 10 μm scale bar were used for analysis. For each hippocampal region, neuronal cells within a defined 50 μm² area were counted at three randomly selected sites per section using ImageJ software (National Institutes of Health, Bethesda, MD, USA). The mean value from the three selected fields was used in the statistical analysis. Histopathological evaluations were performed by an investigator blinded to treatment allocation.

### Statistical analysis

Data were analyzed using GraphPad Prism version 8 (GraphPad Software Inc., La Jolla, CA, USA) and are presented as mean ± SEM. Group comparisons were performed using one-way or two-way analysis of variance (ANOVA), as appropriate. When significant main effects or interactions were detected, Tukey’s or Bonferroni’s post hoc multiple-comparison tests were applied. Differences were considered statistically significant at p < 0.05.

## RESULTS

### Effect of melatonin on learning and memory in fluoride-exposed rats

**Figure 1:**
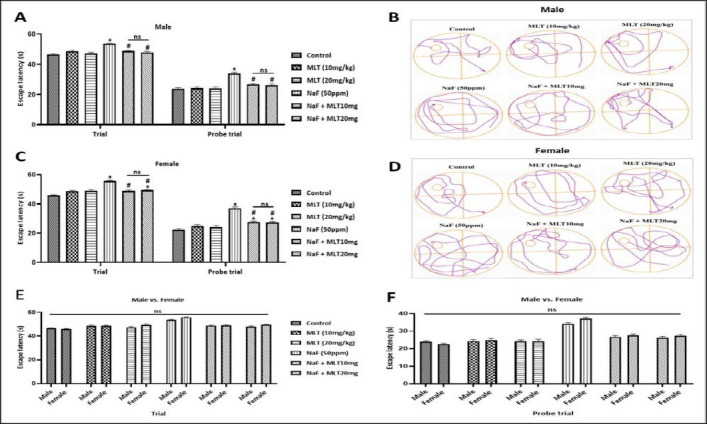
Morris Water Maze (MWM) performance in rats. Escape latency in males (A) and females (C), representative probe trial track plots in males (B) and females (D), and sex comparisons during training (E) and probe trials (F). Data are presented as mean ± standard error of the mean (n = 8 per group). Statistical analysis was performed using two-way analysis of variance followed by Tukey’s multiple-comparison test (A and C) or Bonferroni’s multiple-comparison test (E and F). *p < 0.05 versus control group; #p < 0.05 versus NaF (50 ppm) group; ns = non-significant (p > 0.05).

**Figure 2:**
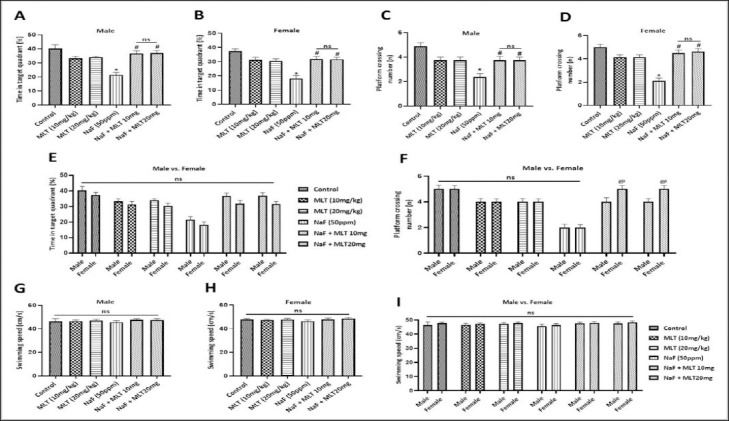
Morris Water Maze performance in rats. Time spent in the target quadrant in (A) males, (B) females, and (E) sex comparisons; platform-crossing numbers in (C) males, (D) females, and (F) sex comparisons; and swimming speed in (G) males, (H) females, and (I) sex comparisons. Data are presented as mean ± standard error of the mean (n = 8 per group). Statistical analysis was performed using one-way analysis of variance (ANOVA) followed by Tukey’s multiple-comparison test (A–D, G, and H) and two-way ANOVA followed by Bonferroni’s multiple-comparison test (E, F, and I). *p < 0.05 versus control group; #p < 0.05 versus NaF (50 ppm) group; ns = non-significant (p > 0.05).

**Figure 3:**
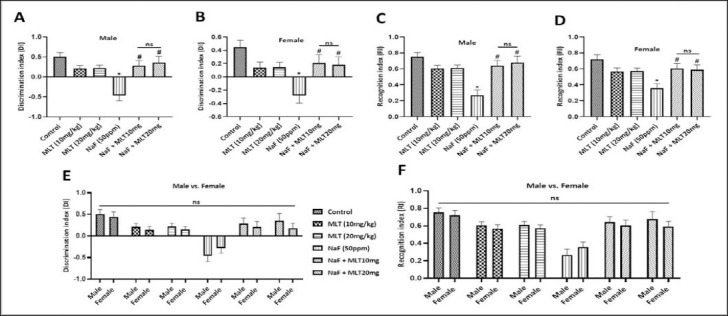
Novel Object Recognition Test performance in rats. Discrimination index in (A) males, (B) females, and (E) sex comparisons, and recognition index in (C) males, (D) females, and (F) sex comparisons. Data are presented as mean ± standard error of the mean (n = 8 per group). Statistical analysis was performed using one-way analysis of variance (ANOVA) followed by Tukey’s multiple-comparison test (A–D) and two-way ANOVA followed by Bonferroni’s multiple-comparison test (E and F). *p < 0.05 versus control group; #p < 0.05 versus NaF (50 ppm) group; ns = non-significant (p > 0.05).

To evaluate the effects of melatonin on learning and memory in fluoride-exposed rats, the animals underwent MWM and NORT assessments (Figures 1–3). Both male and female rats exposed to NaF (50 ppm) exhibited significantly impaired learning and memory performance compared with their respective control groups (p < 0.05). These impairments were evidenced by increased escape latency (Figure 1A and C), decreased time spent in the target quadrant (Figures 2A and B), platform-crossing numbers (Figures 2C and D), DI (Figures 3A and B), and RI (Figures 3C and D).

Co-treatment with melatonin (10 mg/kg and 20 mg/kg) significantly attenuated these fluoride-induced deficits in both sexes (p < 0.05), as evidenced by reduced escape latency and increased time spent in the target quadrant, platform-crossing numbers, DI, and RI. However, no significant differences were observed between the two melatonin doses (p > 0.05). Swimming speed remained comparable among all groups in both sexes, with no significant differences detected (p > 0.05; Figure 2G and H).

Sex-based comparisons revealed no significant differences for most behavioral parameters (p > 0.05). The only exception was platform-crossing number in the MWM test, where females exhibited significantly higher values than males in the NaF + MLT 10 mg/kg and NaF + MLT 20 mg/kg groups (p < 0.05; Figures 1E and F, 2E, F, and I, and 3E and F).

### Effect of melatonin on serum fluoride concentrations and brain SIRT1 levels in fluoride-exposed rats

**Figure 4:**
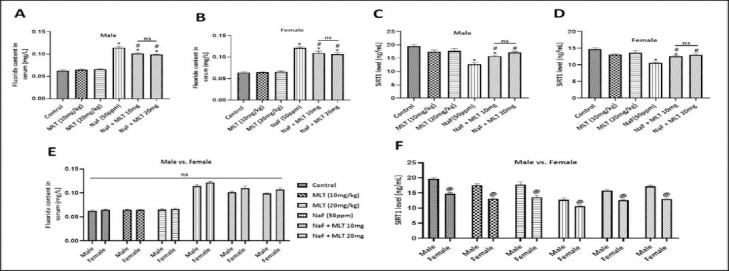
Effect of melatonin on serum fluoride and brain SIRT1 concentrations in rats. Serum fluoride concentrations in (A) males, (B) females, and (E) sex comparisons (n = 6 per group), and brain SIRT1 concentrations in (C) males, (D) females, and (F) sex comparisons (n = 3 per group). Data are presented as mean ± standard error of the mean. Statistical analysis was performed using one-way analysis of variance (ANOVA) followed by Tukey’s multiple-comparison test (A–D) and two-way ANOVA followed by Bonferroni’s multiple-comparison test (E and F). *p < 0.05 versus control group; #p < 0.05 versus NaF (50 ppm) group; ns = non-significant (p > 0.05).

Serum fluoride concentrations were significantly elevated in NaF-treated groups compared with their respective control groups in both sexes (p < 0.05; Figures 4A and B). Administration of melatonin at both doses significantly reduced serum fluoride concentrations relative to fluoride-treated animals. However, no significant differences were observed between the two melatonin doses (p > 0.05). No sex-related differences were detected in serum fluoride concentrations across treatment groups (p > 0.05; Figure 4E).

Fluoride exposure significantly reduced brain SIRT1 concentrations in both male and female rats compared with their respective controls (p < 0.05; Figures 4C and D). Co-treatment with melatonin significantly increased brain SIRT1 concentrations in both sexes (p < 0.05). Females exhibited significantly lower SIRT1 concentrations than males across treatment groups (p < 0.05; Figure 4F).

### Effect of melatonin on oxidative stress and apoptosis markers in fluoride-exposed rats

**Figure 5:**
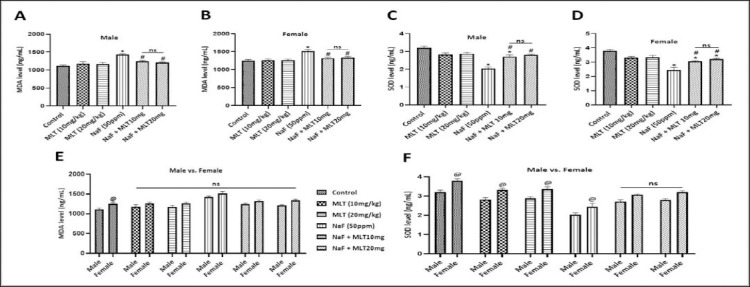
Effect of melatonin on oxidative stress markers in rat brain tissue. Malondialdehyde (MDA) concentrations in (A) males, (B) females, and (E) sex comparisons, and superoxide dismutase (SOD) concentrations in (C) males, (D) females, and (F) sex comparisons. Data are presented as mean ± standard error of the mean (n = 3 per group). Statistical analysis was performed using one-way analysis of variance (ANOVA) followed by Tukey’s multiple-comparison test (A–D) and two-way ANOVA followed by Bonferroni’s multiple-comparison test (E and F). *p < 0.05 versus control group; #p < 0.05 versus NaF (50 ppm) group; ns = non-significant (p > 0.05).

**Figure 6:**
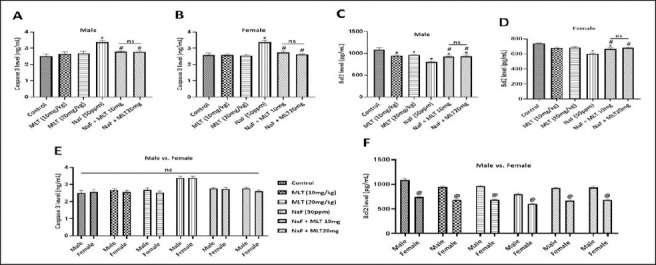
Effect of melatonin on apoptosis markers in rat brain tissue. Caspase-3 concentrations in (A) males, (B) females, and (E) sex comparisons, and B-cell lymphoma 2 (Bcl-2) concentrations in (C) males, (D) females, and (F) sex comparisons. Data are presented as mean ± standard error of the mean (n = 3 per group). Statistical analysis was performed using one-way analysis of variance (ANOVA) followed by Tukey’s multiple-comparison test (A–D) and two-way ANOVA followed by Bonferroni’s multiple-comparison test (E and F). *p < 0.05 versus control group; #p < 0.05 versus sodium fluoride (NaF) (50 ppm) group; ns = non-significant (p > 0.05).

NaF exposure significantly increased brain MDA and caspase-3 concentrations while decreasing SOD and Bcl-2 concentrations in both sexes compared with control animals (p < 0.05; Figures 5 and 6). Co-treatment with melatonin significantly reduced MDA and caspase-3 concentrations and increased SOD and Bcl-2 concentrations in both sexes (p < 0.05). No significant differences were observed between the two melatonin doses.

Sex-based comparisons revealed variable findings. Females in the control group exhibited significantly higher MDA concentrations than males (p < 0.05), whereas no significant differences were observed in other treatment groups. SOD concentrations were significantly higher in females than males in the control, MLT 10 mg/kg, MLT 20 mg/kg, and NaF 50 ppm groups (p < 0.05). However, no sex-related differences were observed in the NaF + MLT 10 mg/kg and NaF + MLT 20 mg/kg groups. Caspase-3 concentrations did not differ significantly between sexes, whereas Bcl-2 concentrations were significantly lower in females across all groups (p < 0.05).

### Effect of melatonin on inflammatory cytokines in fluoride-exposed rats

**Figure 7:**
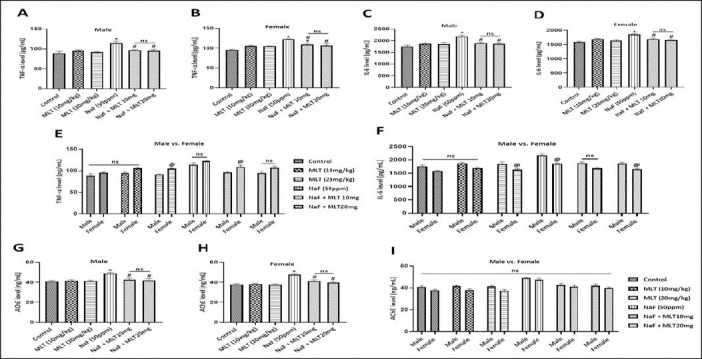
Effect of melatonin on inflammatory cytokines and acetylcholinesterase (AChE) concentrations in rat brain tissue. tumor necrosis factor-α concentrations in (A) males, (B) females, and (E) sex comparisons; interleukin-6 (IL-6) concentrations in (C) males, (D) females, and (F) sex comparisons; and AChE concentrations in males (G), females (H), and sex comparisons (I). Data are presented as mean ± standard error of the mean (n = 3 per group). Statistical analysis was performed using one-way analysis of variance (ANOVA) followed by Tukey’s multiple-comparison test (A–D, G, and H) and two-way ANOVA followed by Bonferroni’s multiple-comparison test (E, F, and I). *p < 0.05 versus control group; #p < 0.05 versus sodium fluoride (NaF) (50 ppm) group; ns = non-significant (p > 0.05).

Fluoride exposure significantly increased brain TNF-α and IL-6 concentrations in both male and female rats compared with their respective control groups (p < 0.05; Figures 7A–D). Co-treatment with melatonin (10 mg/kg and 20 mg/kg) significantly attenuated these increases in both sexes (p < 0.05), with no significant differences observed between the two melatonin doses (p > 0.05).

Sex-based comparisons demonstrated that TNF-α concentrations were significantly higher in females than males in the MLT 20 mg/kg and NaF + MLT 10 mg/kg groups (p < 0.05), whereas no significant differences were observed in the remaining groups (Figure 7E). In contrast, IL-6 concentrations were significantly lower in females than males in the MLT 20 mg/kg, NaF 50 ppm, and NaF + MLT 20 mg/kg groups (p < 0.05), while no significant differences were detected in the other treatment groups (Figure 7F).

### Effect of melatonin on brain AChE concentrations in fluoride-exposed rats

Fluoride exposure significantly increased brain AChE concentrations in both sexes compared with their respective control groups (p < 0.05; Figures 7G and H). Co-treatment with melatonin at both doses significantly reduced AChE concentrations relative to fluoride-treated animals (p < 0.05). No significant differences were observed between males and females in any treatment group (p > 0.05; Figure 7I).

### Effect of melatonin on hippocampal histopathology in fluoride-exposed rats

**Figure 8:**
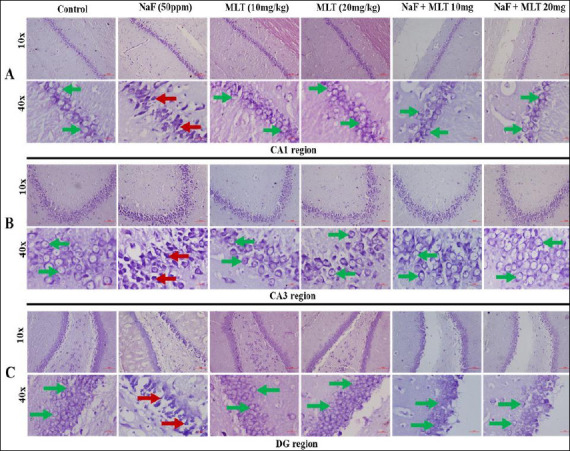
Representative photomicrographs of cresyl violet-stained hippocampal neurons in male rats [objective magnification: 10× (scale bar = 10 μm) and 40× (scale bar = 2 μm)]. (A) CA1 neurons, (B) CA3 neurons, and (C) DG neurons. Green arrows indicate normal neurons, whereas red arrows indicate degenerating neurons.

**Figure 9:**
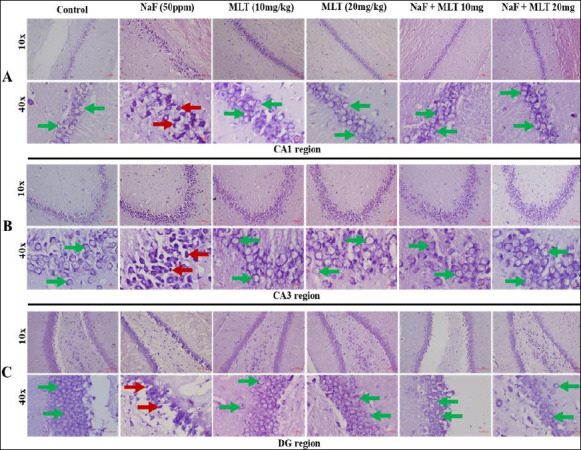
Representative photomicrographs of cresyl violet-stained hippocampal neurons in female rats [objective magnification: 10× (scale bar = 10 μm) and 40× (scale bar = 2 μm)]. (A) CA1 neurons, (B) CA3 neurons, and (C) DG neurons. Green arrows indicate normal neurons, whereas red arrows indicate degenerating neurons.

Cresyl violet (Nissl) staining revealed normal hippocampal morphology in control male and female rats. Neurons within the CA1, CA3, and DG regions exhibited intact plasma membranes, clear cytoplasm, and prominent nuclei, indicative of normal cellular architecture (green arrows; Figures 8A–C and 9A–C).

In contrast, NaF-treated animals displayed marked histopathological alterations in all hippocampal regions examined. Numerous degenerating and pyknotic neurons with flame-shaped basophilic cell bodies were observed in both pyramidal neurons of the CA1 and CA3 regions and granule cells of the DG region (red arrows; Figures 8A–C and 9A–C).

Co-treatment with melatonin (10 mg/kg and 20 mg/kg) substantially preserved neuronal morphology in both sexes. The majority of hippocampal neurons retained normal structural characteristics comparable to those observed in control animals, indicating attenuation of fluoride-induced neuronal damage.

**Figure 10:**
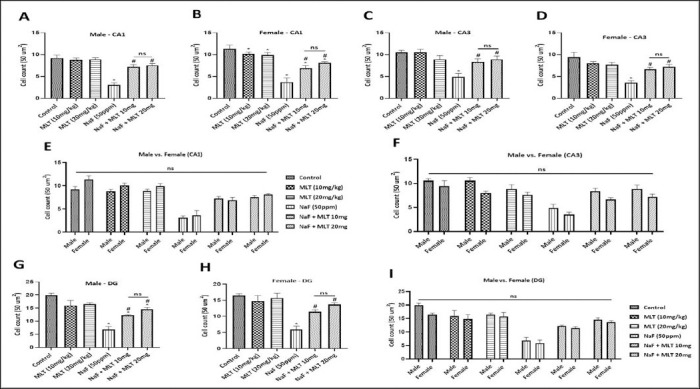
Neuronal cell counts in hippocampal regions of rats. CA1 region in (A) males, (B) females, and (E) sex comparisons; CA3 region in (C) males, (D) females, and (F) sex comparisons; and DG region in males (G), females (H), and sex comparisons (I). Data are presented as mean ± standard error of the mean (n = 3 per group). Statistical analysis was performed using one-way analysis of variance (ANOVA) followed by Tukey’s multiple-comparison test (A–D, G, and H) and two-way ANOVA followed by Bonferroni’s multiple-comparison test (E, F, and I). *p < 0.05 versus control group; #p < 0.05 versus NaF (50 ppm) group; ns = non-significant (p > 0.05).

Quantitative analysis demonstrated a significant reduction in neuronal cell numbers within the CA1, CA3, and DG regions in NaF-treated animals compared with their respective control groups (p < 0.05; Figures 10A–D and G–H). Melatonin co-treatment significantly increased neuronal cell counts relative to fluoride-treated animals in all hippocampal regions examined (p < 0.05). However, no significant differences were observed between the two melatonin doses.

No significant sex-related differences were detected in neuronal cell counts across any hippocampal region or treatment group (p > 0.05; Figures 10E, F, and I).

### Comparison of melatonin doses

No significant differences were observed between the 10 mg/kg and 20 mg/kg melatonin treatment groups for any of the behavioral, biochemical, or histopathological parameters evaluated in either sex (Figures 1–10).

### Sex-related differences in study outcomes

Overall, no consistent sex-related differences were observed across the evaluated behavioral, biochemical, and histopathological parameters. Although significant differences between males and females were detected for selected variables, these findings did not follow a consistent pattern across treatment groups or outcome measures (Figures 1–10).

## DISCUSSION

### Melatonin modulates systemic fluoride burden and restores brain SIRT1 levels

In the current study, significantly elevated serum fluoride concentrations were observed in NaF-exposed male and female rats, which are widely recognized as an important indicator of systemic fluoride exposure and fluorosis [2, 56]. Melatonin co-treatment was associated with a significant reduction in serum fluoride concentrations. Although the mechanism underlying this observation remains unclear, it may reflect alterations in systemic fluoride handling, including potential effects on fluoride absorption, distribution, metabolism, or excretion. Because urinary fluoride excretion was not evaluated in the present study, the contribution of renal clearance could not be determined. Similarly, the possibility that melatonin influences gastrointestinal fluoride absorption remains speculative. Therefore, future studies incorporating pharmacokinetic analyses and fluoride balance assessments are warranted to clarify the mechanisms responsible for the observed reduction in circulating fluoride concentrations.

Consistent with previous reports, the present study’s findings support the concept that SIRT1 downregulation contributes to the pathogenesis of fluoride-induced neurotoxicity and cognitive impairment [39, 57]. SIRT1 is a critical regulator of neuronal survival, neurogenesis, synaptic plasticity, mitochondrial function, and memory formation [58]. In the present study, fluoride exposure significantly reduced brain SIRT1 concentrations in both sexes, whereas melatonin co-treatment restored SIRT1 levels toward control values. These findings suggest a potential association between melatonin-mediated neuroprotection and restoration of SIRT1 signaling. However, because only SIRT1 protein concentrations were measured, the observed relationship remains associative rather than mechanistic. Additional studies evaluating SIRT1 activity, downstream signaling pathways, and functional modulation are required to establish causality.

### Melatonin attenuates oxidative stress, apoptosis, and neuroinflammation

The present study demonstrated that fluoride exposure induced substantial oxidative stress, apoptotic activity, and neuroinflammation, as evidenced by increased MDA, caspase-3, TNF-α, and IL-6 concentrations together with reduced SOD and Bcl-2 concentrations. These findings are consistent with extensive evidence indicating that oxidative stress is a central initiating event in fluoride-induced neurotoxicity, leading to the activation of inflammatory pathways, mitochondrial dysfunction, and neuronal injury [59].

Melatonin co-treatment significantly attenuated these pathological alterations. Animals receiving melatonin exhibited reduced lipid peroxidation, diminished inflammatory cytokine production, and decreased apoptotic signaling, accompanied by restoration of antioxidant defenses. These observations support previous reports describing melatonin as a potent antioxidant and anti-inflammatory molecule capable of protecting neuronal tissues from oxidative injury [23–31].

Although restoration of SIRT1 concentrations coincided with improvements in oxidative stress, apoptosis, and inflammation, the present findings do not establish a direct causal relationship among these events. Importantly, downstream molecular targets commonly associated with SIRT1-mediated neuroprotection were not evaluated. Consequently, whether the observed neuroprotective effects were directly mediated through SIRT1 signaling remains uncertain.

Recent studies have demonstrated that melatonin exerts neuroprotective actions through multiple molecular pathways, including mitophagy-related mechanisms and mitochondrial quality-control systems [32, 33]. Therefore, the neuroprotective effects observed in the present study are likely mediated through several interconnected pathways rather than a single molecular target. SIRT1 may represent one component of a broader neuroprotective network involving oxidative stress regulation, mitochondrial homeostasis, apoptosis suppression, and inflammatory control. This interpretation is further supported by studies demonstrating beneficial effects of other SIRT1-modulating compounds, including resveratrol, in experimental models of fluoride-induced neurotoxicity [20, 21].

### Melatonin improves cognitive function and preserves hippocampal integrity

The hippocampus is a critical brain region involved in learning, memory consolidation, and spatial navigation and is particularly vulnerable to oxidative stress, chronic inflammation, and neurotoxic insults [60, 61]. Histopathological examination in the present study revealed pronounced neuronal degeneration in the CA1, CA3, and DG regions of fluoride-exposed rats. These structural abnormalities were accompanied by significant impairments in MWM and NORT performance, indicating deficits in both spatial and recognition memory.

These findings are consistent with previous studies demonstrating that chronic fluoride exposure disrupts hippocampal architecture and impairs cognitive function [56, 62]. The close correspondence between histopathological damage and behavioral deficits observed in the present study further supports the role of hippocampal dysfunction in fluoride-induced cognitive impairment.

Importantly, melatonin co-treatment markedly preserved hippocampal morphology and significantly improved behavioral performance. The restoration of neuronal integrity in the CA1, CA3, and DG regions was accompanied by improvements in learning and memory parameters, suggesting that structural preservation of the hippocampus contributed to the observed functional recovery. These findings reinforce the therapeutic potential of melatonin as a neuroprotective agent against fluoride-induced hippocampal injury.

### Melatonin attenuates cholinergic dysfunction associated with fluoride exposure

In addition to oxidative and inflammatory mechanisms, the present findings suggest that cholinergic dysfunction contributes to fluoride-induced neurotoxicity. Fluoride exposure significantly increased brain AChE concentrations, a change that may reduce acetylcholine availability and impair cognitive processing [18, 63]. Alterations in cholinergic neurotransmission have been implicated in learning and memory deficits associated with several neurodegenerative and neurotoxic conditions.

Melatonin co-treatment significantly reduced AChE concentrations in fluoride-exposed animals. These findings are consistent with previous reports demonstrating beneficial effects of melatonin on cholinergic function in experimental models of lipopolysaccharide-induced neuroinflammation, scopolamine-induced amnesia, and Alzheimer’s disease [64–66]. Although the precise mechanism remains unclear, the results suggest that the preservation of cholinergic neurotransmission may represent an additional component of melatonin’s neuroprotective profile.

### Influence of sex on fluoride-induced neurotoxicity and melatonin responsiveness

An important aspect of the present study was the inclusion of both male and female animals to evaluate potential sex-related differences in fluoride-induced neurotoxicity and treatment responsiveness. Overall, fluoride exposure produced broadly comparable behavioral, biochemical, and histopathological outcomes in both sexes. Although several statistically significant differences were observed between males and females for selected parameters, these differences were inconsistent and did not indicate a clear sex-specific pattern.

These findings differ somewhat from observations in certain human cohort studies, which have reported greater susceptibility to fluoride-associated neurodevelopmental effects in males [10, 11]. The absence of pronounced sex-related differences in the present study may suggest that the primary mechanisms underlying fluoride-induced neurotoxicity, including oxidative stress, apoptosis, neuroinflammation, and SIRT1-associated signaling, operate similarly in both sexes under the experimental conditions employed.

Nevertheless, an important limitation should be acknowledged. Estrous cycles were not monitored in female rats. Consequently, the potential influence of hormonal fluctuations on behavioral and biochemical outcomes cannot be excluded. Future studies incorporating estrous cycle monitoring, hormonal profiling, and larger sample sizes may provide additional insights into sex-dependent responses to fluoride exposure and melatonin treatment.

### Dose-response considerations and future perspectives

A notable finding of the present study was the absence of significant differences between the 10 mg/kg and 20 mg/kg melatonin treatment groups across behavioral, biochemical, and histopathological outcomes. This observation suggests that the lower dose may have been sufficient to achieve near-maximal neuroprotective effects under the experimental conditions employed, with limited additional benefit from dose escalation.

From a translational perspective, the lower dose may be advantageous because lower dose interventions are generally associated with reduced treatment costs and potentially improved clinical feasibility. However, the doses used in the present study are experimental and intended to explore mechanistic effects rather than to establish direct clinical equivalence. Therefore, future investigations should evaluate a broader dose range, pharmacokinetic profiles, and clinically relevant dosing regimens to improve translational applicability.

## CONCLUSION

The present study demonstrated that chronic fluoride exposure induced significant neurotoxicity and cognitive impairment in both male and female Wistar rats. Fluoride exposure increased serum fluoride concentrations and was associated with reduced brain SIRT1 levels, enhanced oxidative stress, apoptosis, neuroinflammation, cholinergic dysfunction, and marked histopathological alterations in the hippocampus. These molecular and structural changes were accompanied by impaired learning and memory performance, as evidenced by deficits in MWM and NORT outcomes.

Melatonin administration at both 10 mg/kg and 20 mg/kg effectively attenuated the adverse effects of fluoride exposure. Melatonin reduced serum fluoride concentrations, restored brain SIRT1 levels, improved antioxidant status, suppressed inflammatory and apoptotic responses, normalized AChE concentrations, preserved hippocampal neuronal architecture, and significantly improved cognitive performance. These findings suggest that melatonin exerts broad neuroprotective effects against fluoride-induced neuronal injury by modulating multiple interconnected pathways involved in oxidative stress, inflammation, apoptosis, and neuronal survival.

A major strength of this study was the comprehensive evaluation of behavioral, biochemical, and histopatho-logical parameters, together with the assessment of SIRT1 levels in both sexes. The inclusion of male and female animals enabled evaluation of potential sex-related differences and demonstrated that fluoride-induced neurotoxicity and melatonin-mediated protection were largely comparable between sexes under the experimental conditions employed.

Several limitations should be acknowledged. The study assessed only SIRT1 protein concentrations and did not evaluate SIRT1 activity or downstream signaling pathways. Urinary fluoride excretion, pharmacokinetic parameters, mitochondrial function, and mitophagy-related markers were not investigated. In addition, estrous cycles were not monitored in female animals, limiting interpretation of potential hormonal influences on treatment responses.

From a practical perspective, the comparable efficacy of the two melatonin doses suggests that a lower melatonin dose may be sufficient to achieve substantial neuroprotective benefits, which could be relevant to future preventive or therapeutic strategies in populations exposed to excessive fluoride. Given melatonin’s affordability, accessibility, and favorable safety profile, it may represent a promising adjunctive approach to mitigating fluoride-associated neurological damage.

Future studies should investigate SIRT1 activity, downstream molecular targets, mitochondrial and mitophagy-related pathways, fluoride pharmacokinetics, and clinically translatable melatonin dosing regimens. Such investigations will help clarify the precise mechanisms underlying melatonin-mediated neuroprotection and strengthen the translational relevance of these findings.

In conclusion, melatonin effectively mitigated fluoride-induced neurotoxicity and cognitive dysfunction in Wistar rats by reducing oxidative stress, neuroinflammation, apoptosis, cholinergic dysfunction, and hippocampal damage, and by restoring SIRT1 levels. These findings support melatonin’s potential as a neuroprotective agent against chronic fluoride exposure and provide a foundation for future mechanistic and translational investigations.

## DATA AVAILABILITY

All data generated during this study are included in this manuscript. Further inquiries can be directed to the corresponding author.

## AUTHORS’ CONTRIBUTIONS

NB: Conceptualization, methodology, investigation, data collection, visualization, and writing – original draft preparation. NK and SGD: Conceptualization, supervision, and writing – review and editing. AK, KGMR, and GS: Formal analysis and manuscript review. SS: Conceptualization, supervision, project administration, funding acquisition, and writing – review and editing. All authors have read and approved the final manuscript.
